# Gibberellic acid promotes selenium accumulation in *Cyphomandra betacea* under selenium stress

**DOI:** 10.3389/fpls.2022.968768

**Published:** 2022-09-02

**Authors:** Yaxin Xu, Lu Zhang, Jin Wang, Dong Liang, Hui Xia, Xiulan Lv, Qunxian Deng, Xun Wang, Xian Luo, Ming’an Liao, Lijin Lin

**Affiliations:** ^1^Institute of Pomology and Olericulture, Sichuan Agricultural University, Chengdu, China; ^2^College of Horticulture, Sichuan Agricultural University, Chengdu, China

**Keywords:** gibberellin, selenium, *Cyphomandra betacea*, physiology, growth

## Abstract

The selenium (Se) deficiency is threatening the human health, and the increase of Se content in food can prevent the Se deficiency of human body. To increase the Se content in fruit trees and alleviate the Se stress to fruit trees, the effects of gibberellic acid (GA) on the growth and Se accumulation in *Cyphomandra betacea* under Se stress were studied. Although GA increased the biomass of *C. betacea*, it did not significantly affect the root/shoot ratio. The root and shoot biomass had a quadratic polynomial regression relationship with the GA concentration. Furthermore, GA increased the photosynthetic pigment content, photosynthetic parameters, and antioxidant enzyme activity of *C. betacea*. GA also increased the Se content in *C. betacea*, peaking at 300 mg/L GA. For instance, GA (300 mg/L) increased the Se contents in roots and shoots of *C. betacea* by 70.31 and 22.02%, respectively, compared with the control. Moreover, the root Se and shoot Se contents had a quadratic polynomial regression relationship with the GA concentration. Correlation and gray relational analyses showed that the carotenoid, chlorophyll *a*, and chlorophyll *b* contents were closely related to the Se uptake in *C*. *betacea* under the GA application. These results show that GA (300 mg/L) can promote the growth and Se uptake of *C*. *betacea* under Se stress.

## Introduction

Soil selenium (Se) is unevenly distributed worldwide, with the most areas lacking Se (Li et al., 2020). Se is one of the essential trace elements of human body, and the Se deficiency is threatening the human health. Eating the Se-enriched vegetables and fruits can safely and effectively increase the Se content in human body (Jiang et al., 2016). Therefore, the increase of Se content in vegetables and fruits can prevent the Se deficiency of human body (Zhao, 2019; Li et al., 2020). Most crops are non-Se accumulators with the Se content not exceeding 30 mg/kg, which cannot meet the dietary requirements in humans (Zhang et al., 2002). Se content in plants positively correlates with the exogenous Se concentration application (Jin et al., 2003; Wang et al., 2016). Various methods, such as the Se application in soil, Se seed dressing, foliar application of Se on leaves, and Se-containing nutrient solution culture, are mainly used to increase the Se content in crops (Hawkesford and Zhao, 2007; Boldrin et al., 2013). However, these methods have many limitations, such as high cost and environmental pollution (Wu et al., 2015; Chen et al., 2018). Therefore, other methods that can improve the Se uptake ability in crops should be identified.

Se uptake in crops has been improved using agronomic measures, such as intercropping, grafting, and application of plant hormones (Luo, 2019; Huan et al., 2021; Pan et al., 2021). Plant hormones mediate the plant growth and nutrient (including Se) absorption (Peleg and Blumwald, 2011). For instance, gibberellic acid (GA) promotes the photosynthesis, vegetative growth, and fruit development of crops (CastroCamba et al., 2022). GA can regulate the crop growth and promote the nutrient absorption (Hassan and Mansoor, 2017; Zhang et al., 2020). GA produces various responses in plants under stress conditions to improve stress tolerance, thus promoting the growth of plants (Colebrook et al., 2014; Niharika Singh et al., 2020). Moreover, GA can promote the detoxification function of antioxidant enzymes and antioxidant activity of plants to scavenge reactive oxygen species (ROS) under heavy metal stress, thus resisting the adverse reactions of heavy metals (Abolghassem et al., 2020). GA can resist the cadmium (Cd) stress by regulating the expression of various genes related to shoot and root growth, metabolism, photosynthetic pigment, and shoot and root morphology of plants (Rashid et al., 2021). GA could also promote the Cd uptake in accumulator plant *Stellaria media* and improve its phytoremediation capability (Xie et al., 2016). However, GA decreases the Cd uptake in other common plants, thus promoting their growth (Yang et al., 2021). GA promoted the nitrogen uptake and utilization rate in lettuce, thus improving the quality and yield of lettuce (Miceli et al., 2019). Furthermore, GA synthesis-related genes and proteins promoted the absorption and utilization of phosphorus in barley GA-deficient mutants (Gualano et al., 2021). GA can also promote calcium absorption after binding to certain proteins, possibly because the protein-GA complex activates Ca^2+^ channels or promotes the degradation of Ca^2+^ channel repressors through the ubiquitin-proteasome pathway. An unknown auxiliary GA receptor may also be involved in the GA-induced increase in calcium (Takeshi et al., 2018). Therefore, GA can promote the growth of plants and improve the plant stress resistance, thus changing nutrient uptake. However, a few studies have reported on the effect of GA on the Se uptake in plants under Se stress.

*Cyphomandra betacea* is a fruit tree belonging to the Solanaceae family, with the high ornamental and edible value (Kouame et al., 2015). A previous study showed that the Se accumulation capacity is lower in *C. betacea* than in other plants (Lin et al., 2020a). So, GA application may increase the Se accumulation capacity of *C. betacea*, thus improving its commercial value. In this study, the effects of different concentrations of GA on the growth and Se uptake of *C. betacea* were studied under Se stress. This study aimed to determine the best GA concentration that could alleviate the Se stress to *C. betacea*, and promote the growth and Se accumulation of *C. betacea*. This study may provide a reference for producing Se-enriched *C. betacea*.

## Materials and methods

### Materials collections and treatments

In September 2021, the mature seeds of *C. betacea* were collected from a 5-year-old fruiting tree in the Chengdu Campus of Sichuan Agricultural University (30°42’N, 103°51’E). The seeds were air-dried, then sown in 50-hole plug seedling trays (53 cm length × 28 cm width × 10 cm height) containing moist perlite. The seedling trays were placed in the greenhouse at 25°C, relative humidity of 70%, and 10000 Lux for 14 h during the day; and at 20°C, relative humidity of 90%, and 0 Lux for 10 h during the night (Liu et al., 2021). The perlite was irrigated with 1/2 Hoagland solution every 3 days after seed germination. The seedlings were transplanted into the pot after growing to about 15 cm (one month later).

GA (GA_3_) was obtained from Beijing XMJ Scientific Co., Ltd., (Beijing, China).

Se used in this experiment was the analytical pure sodium selenite (Na_2_SeO_3_), and obtained from Nanjing Chemical Reagent Co., Ltd., (Nanjing, China).

### Experimental design

Two uniform *C. betacea* seedlings were transplanted into each plastic pot (10 cm height × 15 cm diameter) filled with perlite in October 2021. The pots were also placed in the greenhouse under the conditions described above. Hoagland solution (100 ml) was irrigated into each pot after transplantation for 7 days. GA solution (0, 100, 200, 300, and 400 mg/L) (Yang et al., 2021) was fully sprayed on both sides of the leaves (10 ml per pot). The spraying was conducted again after 15 days. Each treatment had three replicates (three pot as one repetition, and 45 pots in total). Meanwhile, each pot was irrigated with Hoagland solution (100 ml) containing 0.1 mg L^–1^ Se in the form of Na_2_SeO_3_ (Liu et al., 2019) every 3 days during the whole plant growth. The positions of pots were randomly changed to reduce the influence of marginal effects during the whole growth process. The plants were harvested after 30 days of the first GA treatment to determine the various indicators.

### Determination of indicators

In November 2021, the fifth mature leaf (from the top) of each plant were chosen to measure the net photosynthetic rate (Pn), transpiration rate (Tr), stomatal conductance (Gs), and intercellular CO_2_ concentration (Ci) using a Li-6400 photosynthetic system (LI-COR, Lincoln, NE, United States). The Li-6400 photosynthetic system and measurement time were conducted as the previous report (Li et al., 2022). The same leaves were used to determine the contents of photosynthetic pigments (chlorophyll *a*, chlorophyll *b*, and carotenoid) and antioxidant enzymes [superoxide dismutase (SOD), peroxidase (POD), and catalase (CAT)] activities. The photosynthetic pigment content was determined using the ethanol and acetone extraction method as the previous report (Lin et al., 2020b). Pre-treatments were conducted as the previous report (Lin et al., 2020b) for the analysis of antioxidant enzyme activity. The activities of SOD, POD, and CAT, were determined using the nitroblue tetrazole, guaiacol colorimetric, and potassium permanganate titration methods, respectively (Hao et al., 2004). The plants were harvested, washed, and then dried as the previous report (Li et al., 2022). The root and shoot biomass (dry weight) were measured using an electronic balance with an accuracy of one thousandth. The dry plant samples were finely ground, soaked with the nitrate acid and perchloric acid for 12 h, digested until transparent at a hot plate, and then reduced by the hydrochloric acid. The digested solution was made up to 50 ml using the deionized water, then used to determine the total Se content via a hydride generation-atomic fluorescence spectrometry (AFS-9700, Beijing Haiguang Instrument Co., Ltd., Beijing, China) (Li et al., 2022). The root/shoot biomass (dry weight) ratio and chlorophyll *a*/*b* ratio were calculated. The translocation factor (TF) was calculated as follows: Se content in shoots/Se content in roots (Rastmanesh et al., 2010).

### Statistical analysis

The software SPSS 20.0.0 (IBM, Chicago, IL, United States) was used for all statistical analyses. All data with three biological repetitions were subjected to a normal distribution and homogeneity tests before one-way analysis of variance followed by the Duncan’s Multiple Range Test (*P* < 0.05). The relationship between GA concentration and biomass/Se content was analyzed using regression analysis. Pearson correlation was used to analyze the relationships among all items. The gray relational analysis was performed according to the previous reports (Wang, 2019; Ma et al., 2022).

## Results

### Biomass (dry weight)

**FIGURE 1:**
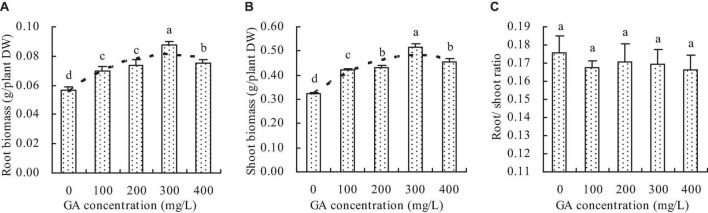
Biomass of *C*. *betacea*. **(A)** Root biomass. **(B)** Shoot biomass. **(C)** Root/shoot ratio. Different lowercase letters indicate significant differences among the treatments (Duncan’s Multiple Range Test, *P* < 0.05).

Compared with the control, GA increased the root and shoot biomass of *C. betacea* (Figures 1A,B). The root and shoot biomass increased with the increase of GA concentration up to 300 mg/L, and then decreased when the GA concentration was higher than 300 mg/L. The root biomass and shoot biomass were got the maximums when the GA concentration was 300 mg/L. Compared with the control, the concentrations of 100, 200, 300, and 400 mg/L GA increased the root biomass by 23.69, 29.65, 54.29, and 33.21%, respectively, and increased the shoot biomass by 29.51, 33.29, 59.51, and 40.46%, respectively. Additionally, the root biomass had a quadratic polynomial regression relationship with the GA concentration (*y* = −2.864E^–7^x^2^ + 0.056, *R*^2^ = 0.814, *P* = 0.000), and the shoot biomass also had a quadratic polynomial regression relationship with the GA concentration (*y* = −1.729E^–6^x^2^ + 0.001x + 0.323, *R*^2^ = 0.860, *P* = 0.001). However, GA did not significantly affect the root/shoot ratio of *C. betacea* (Figure 1C).

### Photosynthetic pigment content

**FIGURE 2:**
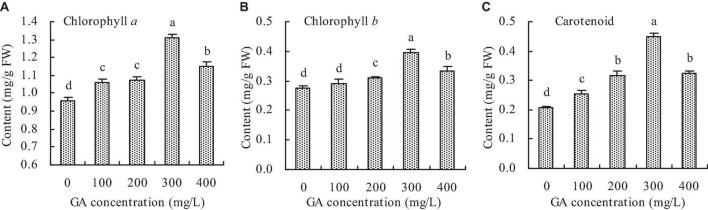
Photosynthetic pigment content in *C. betacea*. **(A)** Chlorophyll *a* content. **(B)** Chlorophyll *b* content. **(C)** Carotenoid content. Different lowercase letters indicate significant differences among the treatments (Duncan’s Multiple Range Test, *P* < 0.05).

Compared with the control, GA increased the chlorophyll *a*, chlorophyll *b*, and carotenoid contents in *C. betacea* to some extent (Figures 2A–C). The contents of chlorophyll *a*, chlorophyll *b*, and carotenoid increased with increasing GA concentration up to 300 mg/L, and then decreased. When the GA concentration was 300 mg/L, the contents of chlorophyll *a*, chlorophyll *b*, and carotenoid were got the maximums, which increased by 36.91, 42.96, and 116.91%, respectively, compared with the control.

### Photosynthetic characteristic

With the increase of GA concentration, the Pn, Gs, Ci, and Tr of *C*. *betacea* increased when the GA concentration was not higher than 300 mg/L, and then decreased when the GA concentration was higher than 300 mg/L (Table 1). GA increased the Pn, Gs, Ci, and Tr to some extent, compared with the control. When the GA concentration was 300 mg/L, the Pn, Gs, Ci, and Tr were got the maximums, which increased by 82.81, 81.01, 30.35, and 72.44%, respectively, compared with the control.

**TABLE 1:** Photosynthetic characteristic of *C*. *betacea*.

GA concentration (mg/L)	Pn (μmol CO_2_/m^2^/s)	Gs (mol H_2_O/m^2^/s)	Ci (μmol CO_2_/mol)	Tr (mmol H_2_O/m^2^/s)
0	4.120 ± 0.097e	0.0437 ± 0.0008c	194.4 ± 4.26d	0.976 ± 0.015e
100	5.209 ± 0.104d	0.0448 ± 0.0019c	209.1 ± 8.25c	1.021 ± 0.011d
200	6.482 ± 0.165c	0.0631 ± 0.0033b	216.1 ± 6.11c	1.362 ± 0.020c
300	7.532 ± 0.117a	0.0791 ± 0.0022a	253.4 ± 9.47a	1.683 ± 0.014a
400	6.835 ± 0.059b	0.0765 ± 0.0016a	232.5 ± 6.49b	1.626 ± 0.021b

Values are means (± SD) of three replicates. Different letters indicate significant differences among the treatments (Duncan’s Multiple Range Test, p < 0.05). Pn, net photosynthetic rate; Gs, stomatal conductance; Ci, intercellular CO_2_ concentration; Tr, transpiration rate.

### Antioxidant enzyme activity

**FIGURE 3:**
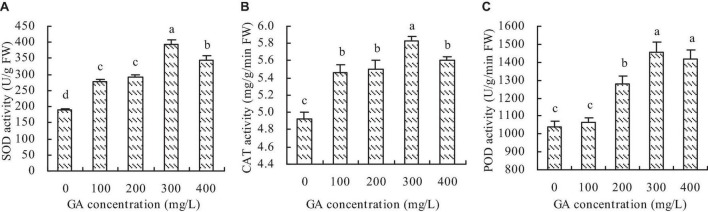
Antioxidant enzyme activity of *C*. *betacea*. **(A)** SOD activity. **(B)** CAT activity. **(C)** POD activity. Different lowercase letters indicate significant differences among the treatments (Duncan’s Multiple Range Test, *P* < 0.05).

Compared with the control, GA increased the SOD, CAT, and POD activities of *C*. *betacea* to some extent (Figures 3A–C). The same as the photosynthetic characteristics, the activities of SOD, CAT, and POD had the increase trend with the increase of GA concentration when the GA concentration was not higher than 300 mg/L, and then had the decrease trend when the GA concentration was higher than 300 mg/L. The maximums of SOD, CAT, and POD activities were at the concentration of 300 mg/L GA, which increased by 107.03, 18.20, and 40.37%, respectively, compared with the control.

### Se content

**FIGURE 4:**
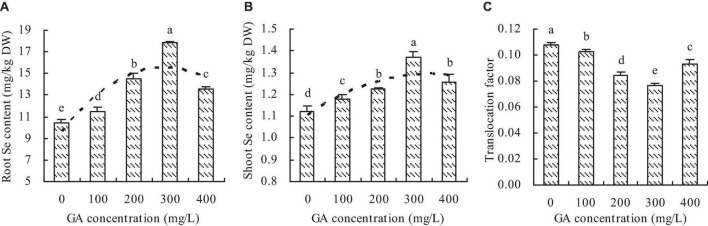
Se content in *C*. *betacea*. **(A)** Root Se content. **(B)** Shoot Se content. **(C)** Translocation factor (TF, shoot Se content/root Se content). Different lowercase letters indicate significant differences among the treatments (Duncan’s Multiple Range Test, *P* < 0.05).

Although GA increased the Se contents in roots and shoots of *C*. *betacea*, the Se contents were higher in roots than in shoots at the different GA concentrations (Figures 4A,B). The Se contents were increased in roots and shoots with increasing GA concentration up to 300 mg/L, then decrease. The maximums of Se contents were at the concentration of 300 mg/L GA. Compared with the control, the concentrations of 100, 200, 300, and 400 mg/L GA increased the root Se content by 9.42, 38.95, 70.31, and 29.39%, respectively, and the shoot Se content by 4.68, 8.83, 22.02, and 11.85%, respectively. Furthermore, the root content had a quadratic polynomial regression relationship with the GA concentration (*y* = −7.391E^–5^x^2^ + 0.042x + 9.583, *R*^2^ = 0.696, *P* = 0.001), and the shoot Se content also had a quadratic polynomial regression relationship with the GA concentration (*y* = −1.655E^–6^x^2^ + 0.001x + 1.105, *R*^2^ = 0.683, *P* = 0.001). GA decreased the TF of C. betacea, and the TF had an opposite trend to the Se content (Figure 4C).

### Correlation and gray relational analyses

**FIGURE 5:**
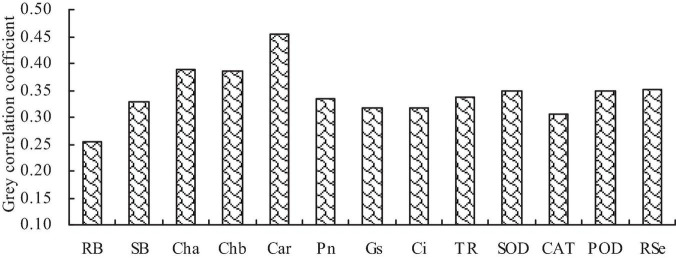
Gray correlation coefficients of biomass, photosynthetic pigment content, photosynthetic characteristic parameter, antioxidant enzyme activity, and root Se content with shoot total Se content. RB, root biomass; SB, shoot biomass; Cha, chlorophyll *a* content; Chb, chlorophyll *b* content; Car, carotenoid content; Pn, net photosynthetic rate; Gs, stomatal conductance; Ci, intercellular CO_2_ concentration; Tr, transpiration rate; SOD, SOD activity; POD, POD activity; CAT, CAT activity; RSe, root total Se content.

Correlation analysis showed that the biomass, Se content, photosynthetic pigment content, photosynthetic characteristic parameter, and antioxidant enzyme activity were significantly positively correlated with each other (Table 2). Gray relational analysis showed that the biomass, photosynthetic pigment content, photosynthetic characteristic parameter, and antioxidant enzyme activity, and root Se content were correlated with the shoot Se content (Figure 5). The order of gray correlation coefficient was as follows: carotenoid content > chlorophyll *a* content > chlorophyll *b* content > root Se content > SOD activity > POD activity > Tr > Pn > shoot biomass > Ci > Gs > CAT activity > root biomass.

**TABLE 2:** Correlations among biomass, Se content, photosynthetic pigment content, photosynthetic characteristic parameter, and antioxidant enzyme activity.

Indicator	Root biomass	Shoot biomass	Chlorophyll *a* content	Chlorophyll *b* content	Carotenoid content	Pn	Gs	Ci	Tr	SOD activity	CAT activity	POD activity	Root Se content	Shoot Se content
Root biomass														
Shoot biomass	0.954**													
Chlorophyll a content	0.936**	0.931**												
Chlorophyll b content	0.881**	0.857**	0.955**											
Carotenoid content	0.923**	0.927**	0.961**	0.960**										
Pn	0.933**	0.940**	0.898**	0.869**	0.923**									
Gs	0.817**	0.832**	0.842**	0.862**	0.870**	0.942**								
Ci	0.893**	0.899**	0.924**	0.945**	0.933**	0.894**	0.892**							
TR	0.840**	0.849**	0.857**	0.872**	0.884**	0.950**	0.994**	0.901**						
SOD activity	0.948**	0.975**	0.939**	0.897**	0.926**	0.949**	0.889**	0.956**	0.907**					
CAT activity	0.965**	0.959**	0.885**	0.792**	0.844**	0.923**	0.793**	0.847**	0.812**	0.943**				
POD activity	0.833**	0.823**	0.841**	0.876**	0.879**	0.930**	0.960**	0.875**	0.974**	0.883**	0.792**			
Root Se content	0.914**	0.889**	0.899**	0.919**	0.969**	0.913**	0.848**	0.883**	0.860**	0.872**	0.830**	0.852**		
Shoot Se content	0.907**	0.924**	0.953**	0.945**	0.954**	0.911**	0.869**	0.899**	0.880**	0.914**	0.866**	0.872**	0.938**	

**Correlation is significant at the 0.01 level (two-tailed test). N = 15. Pn, net photosynthetic rate; Gs, stomatal conductance; Ci, intercellular CO_2_ concentration; Tr, transpiration rate.

## Discussion

Gibberellin participates in various plant life activities and is closely related to the metabolic regulation of plant growth and development (Gao and Fu, 2018). Previous studies have shown that appropriate concentrations of GA could increase the growth of stem length, stem thickness, and leaf area of plum, tomato, and sweet pepper seedlings (Tahani et al., 2019; Miceli et al., 2020). In this experiment, GA increased the root and shoot biomass of *C*. *betacea* under Se stress, which is consistent with the previous studies (Tahani et al., 2019; Miceli et al., 2020). Additionally, the biomass of root and shoot had a quadratic polynomial regression relationship with the GA concentration. This could be due to: First, GA directly regulates the content of aspartic acid–glutamic acid–leucine–leucine–alanine (DELLA) protein in the plant nucleus, thus promoting the plant growth via ubiquitination and degradation of DELLA protein by binding to the specific receptor gibberellin insensitive dwarf 1 (GID1). DELLA protein is a key regulator in the GA signaling pathway, inhibiting the expression of downstream genes, thus inhibiting the elongation of plant hypocotyls and stems (Bai et al., 2019; Jorge et al., 2020). The exogenous application of GA increases the concentration of extracellular active GA in plants, thus enhancing the degradation of DELLA protein (Bai et al., 2019). Second, GA interacts with other plant hormones, thus indirectly affecting the plant growth by changing the levels of auxin, brassinolide, and ethylene (Wang et al., 2017; CastroCamba et al., 2022).

Chlorophyll and carotenoid participate in photosynthesis by capturing light energy, driving electron transfer to reaction centers, and defending against the photo damage (Petra et al., 2003). Furthermore, the photosynthetic carbon assimilation ability of plants is closely related to the photosynthetic traits. Therefore, the photosynthetic indicators can reflect the growth status and productivity of plants (Sun et al., 2017). Under Se stress, the contents of chlorophyll *a*, chlorophyll *b*, and carotenoid in *C*. *betacea* decreased, and had a decrease trend with the increase of Se concentration (Lin et al., 2020a). Studies have shown that a high concentration (over 40 mg/L) of GA decreased the chlorophyll *a* and carotenoid contents, Pn, Ci, Gs, and Tr in spinach seedling leaves, while a low concentration (less than 40 mg/L) of GA had the opposite effect under copper stress (Gong et al., 2021). Under Cd stress, GA also increased the photosynthetic pigment content and photosynthetic parameters of *C*. *betacea* (Yang et al., 2021). In this study, the contents of chlorophyll *a*, chlorophyll *b*, and carotenoid in leaves of *C*. *betacea* increased with increasing GA concentration up to 300 mg/L, and then decreased under Se stress. Moreover, the changes in the Pn, Ci, Gs, and Tr of *C*. *betacea* were similar to the photosynthetic pigment content. These results are consistent with the previous studies (Gong et al., 2021; Yang et al., 2021), indicate that the GA could promote the synthesis of photosynthetic pigments and improve the photosynthesis of *C*. *betacea* under Se stress. Because the GA can promote the transduction of GA signal in plants, up-regulates the expression of photosynthetic pigment synthesis-related genes, and down-regulates the expression of photosynthetic pigment metabolism-related genes, thereby increasing the photosynthetic pigment content (Keawmanee et al., 2022).

Under stress condition, the excessive ROS are produced in plants, which may damage the cellular components, disrupt cell membranes, and inhibit the growth of plants (Labanowska et al., 2012). Se is similar to toxic metals, which also causes the excessive ROS to inhibit the growth of plants (Spallholz and Hoffman, 2002). Under Se stress, the POD and CAT activities of *C*. *betacea* increased, and had an increase trend with the increase of Se concentration (Lin et al., 2020a). Previous studies have shown that an appropriate concentration of GA increased the antioxidant enzyme activity of spinach under copper stress and okra under NaCl stress (Wang et al., 2019; Gong et al., 2021). GA also increased the SOD, POD, and CAT activities of *C*. *betacea* under Cd stress (Yang et al., 2021). Herein, GA increased the SOD, POD, and CAT activities in the leaves of *C*. *betacea* under Se stress, which is consistent with the previous studies (Wang et al., 2019; Gong et al., 2021; Yang et al., 2021). These results indicate that GA could improve the resistance of *C*. *betacea* to Se stress, which may be related to the expressions of antioxidative enzyme-related genes regulating by GA (Peng et al., 2022).

Plants mainly absorb Se from soil through their roots. Soil is the main source of Se for plants (Jiang et al., 2016). Besides soil environment, the form of Se also affects the Se absorption in plants. The transport mechanism and efficiency of different valence Se in plants are different (Wang et al., 2014; Jiang et al., 2016). Some studies have assessed the characteristics of plant hormones on the absorption of Se. For instance, indole acetic acid (IAA) could promote the growth of *C*. *betacea* seedlings and increase the Se content and bioconcentration factor in various organs (Huan et al., 2021). Methyl jasmonate could promote the absorption of low-concentration sodium selenate in tea, thus improving the antioxidant effect (Zhou et al., 2012). Herein, GA increased the Se contents in roots and shoots of *C*. *betacea*. In addition, the Se contents in roots and shoots had a quadratic polynomial regression relationship with the GA concentration. These results indicate that GA could promote the Se absorption in *C*. *betacea*. Additionally, the Se content was higher in roots than that in shoots of *C*. *betacea*, indicating that Se is mainly concentrated in roots of *C*. *betacea*. This could be because sodium selenite was the exogenous Se applied. Sodium selenite mainly exists in the form of selenomethionine (organic Se) in plants, which is easily fixed in plant roots (Zhu et al., 2009). Moreover, correlation analysis showed that the biomass, Se content, photosynthetic pigment content, photosynthetic characteristic parameter, and antioxidant enzyme activity were significantly positively correlated with each other. Gray relational analysis also showed that carotenoid, chlorophyll *a*, and chlorophyll *b* contents were the most related to the shoot Se content. These results show that photosynthetic pigment content was closely related to the Se uptake in *C*. *betacea* under the GA application. However, further studies should assess the action mechanisms.

## Conclusion

Under Se stress, GA increased the biomass, photosynthetic pigment content, photosynthetic rate, and antioxidant enzyme activity of *C*. *betacea*, thereby promoting the growth of *C*. *betacea*. GA also increased the Se content in *C. betacea*, peaking at 300 mg/L GA. The biomass and Se content had a quadratic polynomial regression relationship with the GA concentration. Among these indicators, the carotenoid, chlorophyll *a*, and chlorophyll *b* contents were the most related to the shoot Se content. The future work should assess the mechanism underlying GA promoting effects on the Se uptake in *C*. *betacea*.

## Data availability statement

The original contributions presented in this study are included in the article/supplementary material, further inquiries can be directed to the corresponding author.

## Author contributions

YX, LZ, and LL conceived and designed the research and checked and revised the manuscript. JW, DL, HX, XLv, and QD performed the experiments. XW, XLu, and ML analyzed the data. YX prepared and wrote the manuscript. All authors contributed to the article and approved the submitted version.
